# Krüppel-Like Factor 8 Is a New Wnt/Beta-Catenin Signaling Target Gene and Regulator in Hepatocellular Carcinoma

**DOI:** 10.1371/journal.pone.0039668

**Published:** 2012-06-27

**Authors:** Tian Yang, Sheng-Yun Cai, Jin Zhang, Jun-Hua Lu, Chuan Lin, Jian Zhai, Meng-Chao Wu, Feng Shen

**Affiliations:** 1 Department of Hepatic Surgery, Eastern Hepatobiliary Surgery Hospital, Second Military Medical University, Shanghai, China; 2 Department of Gynaecology and Obstetrics, Changhai Hospital, Second Military Medical University, Shanghai, China; The University of Hong Kong, China

## Abstract

Krüppel-like factor 8 (KLF8) plays important role in cell cycle and oncogenic transformation. Here we report the mechanisms by which KLF8 crosstalks with Wnt/β-catenin signaling pathway and regulates hepatocellular carcinoma (HCC) cells proliferation. We show that overexpression of KLF8 and nucleus accumulation of β-catenin in the human HCC samples are positively correlated. More importantly, KLF8 protein levels plus nucleus accumulation of β-catenin levels were significantly elevated in high-grade HCC compared to low-grade HCC. Using HCC HepG2 cells we find that, on the one hand both protein and mRNA of KLF8 are up-regulated under Wnt3a stimulation, on the other hand overexpression of KLF8 increases the cytoplasm and nucleus accumulation of β-catenin, recruits p300 to β-catenin/T-cell factor 4 (TCF4) transcription complex, enhances TOP flash report gene transcription, and induces Wnt/β-catenin signaling target genes c-Myc, cyclin D1 and Axin1 expression. Knockdown of KLF8 using shRNA inhibits Wnt3a induced transcription of TOP flash report gene and expression of c-Myc, cyclin D1 and Axin1. Knockdown of β-catenin by shRNA rescues the enhanced HepG2 and Hep3B cells proliferation ability induced by overexpression of KLF8.

## Introduction

Canonical Wnt/β-catenin signaling pathway plays essential role in regulating developmental decision and adult tissue homeostasis. The key regulatory molecule is β-catenin, which is kept low level in cytoplasm by its binding to the cytoplasmic tail of E-cadherin and its degradation through a destruction complex consisting of Axin1, glycogen synthase kinase-3β (GSK-3β), adenomatous polyposis coli (APC) and casein kinase Iα (CKIα) in the absence of Wnt signaling. Activation of canonical Wnt signaling by Wnt binding to the 7-span transmembrane protein Frizzled (Fz) and the single-span low-density lipoprotein receptor-related protein (LRP) leads to inhibition of GSK-3β activity and translocation of β-catenin to the nucleus, where it interacts with the TCF/lymphoid enhancer factor (LEF) family of transcription factors for up-regulation of such target genes as Axin1, c-Myc and cyclin D1 [1]. Various human cancers including HCC manifest abnormal β-catenin/TCF/LEF transactivation due to mutations in Wnt/β-catenin signaling pathway such as β-catenin, APC, axin, etc. [2]–[7].

KLF8 belongs to the Krüppel-like factor (KLF) family of transcription factors, which share homology in their three C2–H2 zinc finger DNA binding domains, play critical role in diverse processes, including regulation of the cell cycle progression, oncogenic transformation and cell invasion [8]–[10]. KLF8 has been shown to be positively regulated by activation of Focal Adesion Kinase (FAK) and PI3K/Akt signaling pathway [9], [11]. KLF8 has been found over-expressing and playing important roles in many human malignant tumors [10]–[12]. Recent investigation revealed that KLF8 is highly expressed in HCC tissues and promotes HCC cell proliferation and invasion [13]. However, the mechanism how KLF8 regulates HCC cell proliferation is still unknown.

Here, we report that KLF8 is a novel participator in canonical Wnt/β-catenin signaling pathway. We show that activation of Wnt/β-catenin signaling increases the expression of KLF8, in the meanwhile, KLF8 stabilizes β-catenin, binds with β-catenin/TCF4 complex, enhances Wnt/β-catenin signaling transcription activation in HCC cells.

## Materials and Methods

### Cell Cultures, Plasmids, Antibodies and Chemicals

HCC cell lines HepG2 and Hep3B, L Wnt-3A cells and control L cells, were purchased from American Type Culture Collection (ATCC), and were cultured according to the recommendations from ATCC. Wnt-3a conditioned medium and control L cell medium were harvested and treated HepG2 cells for 4 hours for western blot assay, 16 hours for qRT-PCR assay and report gene assay. Human β -catenin shRNA, target sequences: 5′-CTGATATTGATGGACAGTA-3′, and human KLF8 shRNA plasmids, including shRNA1, target sequence: 5′-CTGGTCGATATGGATAAACTCA-3′, shRNA2, target sequence: 5′-CAGCAGATCTTACATGTCA-3′, and shRNA control, target sequence: TGAGCAGGCGCATGTGCTG-3′, were obtained from Open Biosystems. The plasmid of KLF8 (MHS1010-98053352) was purchased from Open Biosystems and was subcloned into pCMV-tag5A vector with c-myc tag. Primary antibodies to β-catenin and cyclin D1 were purchased from BD Biosciences. Primary antibodies to α-tubulin and Sp1 were purchased from Sigma. Anti-c-Myc antibody was purchased from ATCC. Primary antibodies to KLF-8, Axin1, p300, and TCF4 were purchased from Abcam. Second antibodies anti-mouse IgG-HRP and anti-rabbit-HRP were purchased from Sigma. MTT assay reagents were purchased from DingGuo Biotech.

### Immunohistochemical Staining

HCC samples were obtained from Eastern Hepatobiliary Surgery Hospital. 5-µm thick tissues sections were gotten and dewaxed, endogenous peroxidase was quenched by incubating with 3% H_2_O_2_ in methanol for 30 min. After incubating with blocking buffer (10% BSA in TBS) at 37°C for 1 hour, tissues sections were incubated with primary antibodies in TBS containing 1% BSA at 4°C overnight, followed by incubation with a horseradish peroxidase anti-rabbit or –mouse IgG antibody. Color was then developed by incubation with an ImmunoPure Metal Enhanced Diaminobenzidine (DAB) Substrate kit (Pierce). After each incubation, tissue sections were washed three times in TBS over 10 min. Tissue sections were finally counterstained with hematoxylin.

### Immunoblotting and Immunoprecipitation Assay

Cells were washed with ice-cold phosphate buffer saline, pH 7.4 (PBS) and lysed with ice-cold radioimmunoprecipitation assay lysis buffer (50 mM Tris-HCl, pH 7.4, 150 mM NaCl, 1 mM sodium orthovanadate 10 mM sodium fluoride, 1 mM phenylmethylsulfonyl fluoride, 2 µg/ml aprotinin, 2 µg/ml leupeptin, 1 µg/ml pepstatin A, 15 µg/ml benzamidine, 0.5% NP-40, 0.15% bovine serum albumin and 10% glycerol) at 4°C for 1 hrs. Samples were centrifuged at 12,000 *g* for 15 min at 4°C. For subcellular fractionation, cell-surface, cytoplasmic and nuclear extracts were prepared using Qproteome Cell Compartment Kit (Qiagen). Samples were subjected to SDS-PAGE, transferred to PVDF membranes (Millipore) and detected with appropriate primary antibodies followed by horseradish peroxidase-conjugated goat anti-mouse or rabbit IgG. The blotting signals were detected using SuperSignal West Dura Extended Duration Substrate (Pierce). Quantitative analyses of immunoblotting signals were obtained via densitometry analysis using LAS4000 Image Software (Fuji Film).

For immunoprecipitation, 1 µg appropriate antibody was preincubated with 30 µl slurry of Protein A-agarose beads (GE Healthcare Life Sciences). Lysates (∼1 mg/sample) were incubated with the antibody-bound Protein A-agarose beads at 4°C overnight. After extensive washing with the radioimmunoprecipitation assay lysis buffer, samples were resuspended in the reducing SDS sample loading buffer, boiled for 5 min, and subjected to SDS-PAGE and immunoblotting.

### qRT-PCR

Total RNA was extracted using the Absolutely RNA Miniprep Kit (Stratagene) and reverse transcribed using ThermoScript RT-PCR System (Invitrogen). The resulting cDNA was used for PCR using the SYBR-Green Master PCR Mix (Applied Biosystem) in triplicates. All RT^2^ qPCR Primer pairs were purchased from SABiosciences. PCR and data collection were performed on the Mx3000 qPCR System (Stratagene). All quantitations were normalized to an endogenous β-actin control. The relative quantitation value for each target gene compared to the calibrator for that target is expressed as 2-(Ct-Cc) (Ct and Cc are the mean threshold cycle differences after normalizing to β-actin). The relative expression levels of samples are presented using a semi-log plot.

### Luciferase Reporter Assay

To evaluate β-catenin/TCF-dependent transcriptional activity, luciferase reporter assay was performed with a pair of luciferase reporter constructs TOP-FLASH and FOP-FLASH (Upstate Biotechnology). TOP-FLASH contains three copies of the TCF binding sites, and FOP-FLASH contains mutated TCF binding sites. Cells were transiently transfected in triplicate with one of these luciferase reporters and pCMV-β-galactosidase (Promega) using Lipofectamine 2000 (Invitrogen). 48 h after transfection, luciferase activity was determined using the Luciferase Assay System Kit (Promega). β-galactosidase activity was determined using the Luminescent β-gal Detection Kit II (BD Clontech) as an internal control.

### Cell Proliferation MTT Assay

Cells including all transfectants were grown in exponential phase and detached by trypsin treatment. Viable cells (5×10^3^ cells/ml) were plated into 96-well tissue culture plates (100 µl complete medium/well) and cultured at 37°C in 5% CO_2_ atmosphere. At different time points, tetrazolium salt was added (20 µl per well) and incubated at 37°C for 4 hr. The insoluble blue formazan product was solubilized by addition of 100 µl/well 10% SDS/5% isobutanol. The plates were read on a micro-titer plate reader using a test wavelength of 570 nm and a reference wavelength of 630 nm.

### Statistical Analysis

To compute correlation coefficients of immunostaining scores between two proteins, Spearman’s rank correlation coefficient was used. P values of less than 0.05 were considered statistically significant. All computations were made with R 2.9.0 (www.r-project.org).

## Results

### KLF8 Overexpresses in HCC Samples and Stabilizes β-catenin

**Figure 1:**
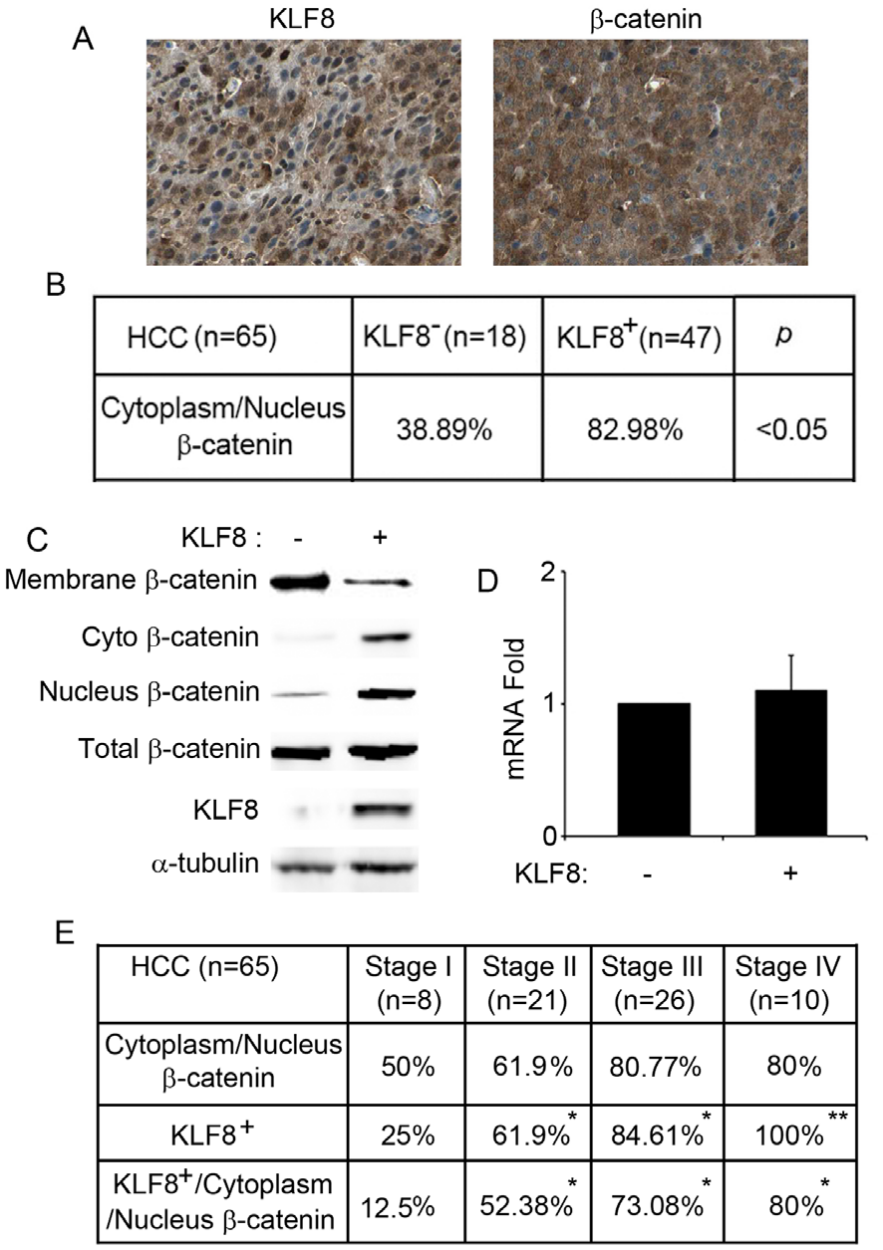
Relative expression of KLF8 and β–catenin in HCC. (A) Immunohistochemical staining of KLF8 and β-catenin in continuous sections of HCC tissues. Results are representative of at least ten separate experiments. (B) Relationships of the KLF8 antigens with cytoplasmic and nucleus β–catenin, in tissue specimens of HCC. Pearson’s Chi-Square test was used for statistical analysis. (C) KLF8 induced β–catenin cytoplasmic and nucleus accumulation in HepG cells. Control pCMV-tag5a vector or pCMV-tag5a/KLF8 plasmid was transfected into HepG2 cells, cell lysates were then analyzed with antibodies. (D) KLF8 did not change mRNA level of β–catenin by qRT-PCR assay. (E) The clinicopathological correlation of KLF8 or/and cytoplasm and nuclear accumulation in HCC samples. *, *p*<0.05, **, *p*<0.01.

We first detected expression pattern of KLF8 protein in human HCC samples using immunohistochemical analysis, immunoreactivity for KLF8 antigens was seen in 72.3% (47/65) of HCC samples. We then examined possible correlation of the positive KLF8 with β-catenin in these 65 cases of HCC by immunohistochemistry assay. Using continuous tissue sectioning, we found that 82.98% (39/47) of KLF8 positive HCC samples showed cytoplasmic and nuclear β-catenin accumulation, compared to only 38.89% (7/18) KLF8 negative HCC samples showed cytoplasmic and nuclear β-catenin accumulation (p<0.05, Fig. 1A, B). In HCC HepG2 cells, we found that, when we overexpressed KLF8, the membrane β-catenin decreased, the cytoplasmic and nuclear β-catenin increased, but the total β-catenin protein and mRNA level did not change (Fig. 1C, D). These results indicate that KLF8 stabilizes β-catenin protein in cytoplasm and nucleus but not increased the transcription of β-catenin gene.

### Expression of KLF8 Correlates with Progressed HCC

We then detected the clinicopathological correlation of KLF8 positive or/and cytoplasm and nuclear β-catenin accumulation in the 65 HCC samples. Expression of KLF8 was detected in 25% (2/8) of Stage I HCC samples, 61.9% (13/21) of Stage II HCC samples, 84.61% (22/26) Stage III HCC samples, and 100% (10/10) Stage IV HCC samples (Fig. 1E), which showed expression of KLF8 positively correlates with progressed HCC. Although cytoplasm and nuclear β-catenin accumulation did not show significant correlations with progressed HCC (50% (4/8) of Stage I HCC samples, 61.9% (13/21) of Stage II HCC samples, 80.77% (21/26) Stage III HCC samples, and 80% (8/10) Stage IV HCC samples), expression of KLF8 plus cytoplasm and nuclear β-catenin accumulation showed positive correlations with progressed HCC (12.5% (1/8) of Stage I HCC samples, 52.38% (11/21) of Stage II HCC samples, 73.08% (19/26) Stage III HCC samples, and 80% (8/10) Stage IV HCC samples, Fig. 1E ).

### Activation of Wnt/β-catenin Signaling Increases the Expression of KLF8

**Figure 2:**
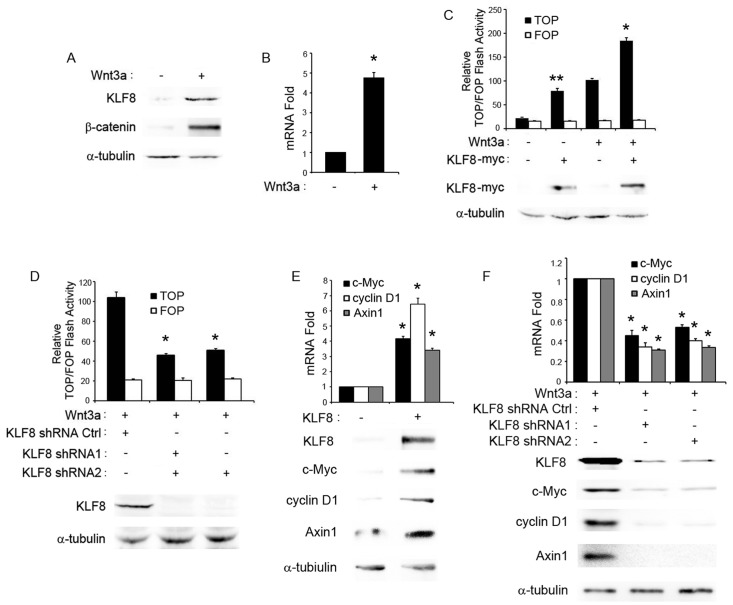
KLF8 is a new Wnt/β–catenin target gene and regulator. (A) KLF8 protein was up-regulated when Wnt3a conditioned medium added, cytoplasmic β–catenin (Cyto-β–catenin) as Wnt3a signaling positive control and α-tubulin as loading control. (B) KLF8 mRNA level was up-regulated when Wnt3a conditioned medium added. (C) KLF8 increased TCF-4 promoter activities (TOP flash) with or without Wnt3a conditioned medium. FOP flash was as negative report gene control. The IB results showed overexpression level of KLF8, α-tubulin as loading control. (D) Knockdown of KLF8 decreased TCF-4 promoter activities (TOP flash) when Wnt3a conditioned medium added. Fop flash was as negative report gene control. The IB results showed down-regulated protein level of KLF8 by two shRNA compared to control shRNA (KLF8 shRNA Ctrl), α-tubulin as loading control. (E) Both mRNA and protein levels of Wnt/β–catenin target gene, c-Myc, cyclin D1 and Axin1, were up-regulated by overexpression of KLF8. (F) Both mRNA and protein levels of Wnt/β–catenin target gene, c-Myc, cyclin D1 and Axin1, were down-regulated by knockdown of KLF8 with shRNA1 or shRNA2 compared to control shRNA (KLF8 shRNA Ctrl). *, *p*<0.05, **, *p*<0.01.

We next asked whether KLF8 was regulated by Wnt/β-catenin signaling pathway. Wnt3a conditioned medium was used to activate Wnt/β-catenin signaling pathway. Compared with control medium, the cytoplasmic β-catenin in HepG2 cells treated with Wnt3a conditioned medium was dramatically increased (Fig. 2A), which demonstrated Wnt/β-catenin signaling pathway was activated. By western blotting assay, we then found that KLF8 protein was increased along with activation of Wnt/β-catenin signaling pathway (Fig. 2A). Using qRT-PCR assay, we found that the mRNA level of KLF8 was also up-regulated by Wnt3a conditioned medium (Fig. 2B). These results indicate KLF8 is a Wnt/β-catenin signaling target gene.

### KLF8 Induces Activation of Wnt/β-catenin Signaling Pathway

We next explored whether KLF8 affect Wnt/β-catenin signaling pathway. Luciferase reporter assay was employed here. HepG2 cells were co-transfected with a plasmid encoding the TCF4 binding sites luciferase reporter (TOP-Flash) or its control reportor (FOP-Flash) and the system control plasmid encoding the β-galactosidase reporter. The results showed that, ectopic expression of KLF8 increased TOP-Flash activity no matter with or without Wnt3a conditioned medium, but not FOP-Flash activity (Fig. 2C). To further test whether KLF8 was necessary for the activation of Wnt/β-catenin signaling, we knockdown the expression of KLF8 in HepG2 cells using shRNA when Wnt3a stimulation. As shown in Fig. 2D, KLF8-shRNA1 and KLF8-shRNA2 decreased the expression of KLF8 compared with control shRNA, in the meanwhile, the TOP-Flash but not FOP-Flash was down-regulated by KLF8-shRNA1 and KLF8-shRNA2, but not control shRNA.

To further investigate whether KLF8 regulated Wnt/β-catenin signaling transcription activity, we examined the expression of Axin1, c-Myc and cyclin D1, which are well characterized targeting genes of Wnt/β-catenin signaling. Indeed, ectopic expression of KLF8 triggered mRNA and protein expression of Axin1, c-Myc and cyclin D1 in HepG2 cells (Fig. 2E), whereas knockdown of KLF8 attenuated their expression in HepG2 cells stimulated with Wnt3a conditioned medium (Fig. 2F). These results clearly indicate that KLF8 participate and is necessary in Wnt/β-catenin signaling pathway for inducing TCF-4 transcription activation, leading to the expression of the Wnt targeting genes, Axin1, c-Myc and cyclin D1.

### KLF8 Recuits P300 to β-catenin/TCF4 Transcription Complex

**Figure 3:**
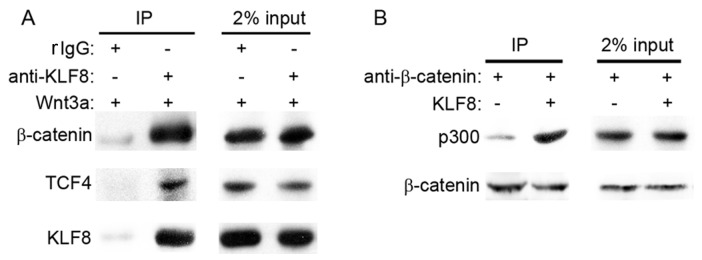
KLF8 recruits P300 to β-catenin/TCF4 transcription complex. (A) KLF8 binding to β-catenin and TCF4. KLF8 was immunoprecipitated from the cell lysates when Wnt3a conditioned medium added, followed by immunoblotting with the antibodies to β-catenin, TCF4 and KLF8. rIgG as immunoprecipitation negative control antibody. Samples loading control were shown (2% input). (B) KLF8 increase P300 binding to β-catenin. β-catenin was immunoprecipitated from the cell lysates transfected with KLF8 or control vector when Wnt3a conditioned medium added, followed by immunoblotting with the antibodies to KLF8 and β-catenin. Samples loading control were shown (2% input).

To investigate how KLF8 regulated Wnt/β-catenin signaling pathway, we first explored whether KLF8 binded with β-catenin. Using co-immunoprecipitation (co-IP) assay, we found that β-catenin could bind with KLF8 in HepG2 cells treated with Wnt3a conditioned medium (Fig. 3A). Importantly, TCF4 was also detected in the co-IP complex, which indicated that KLF8 binded with β-catenin/TCF4 transcription complex. P300 is needed in β-catenin/TCF4 transcription complex activation and also binds with KLF8 [15], [16], we thus suspected whether KLF8 recruits P300 to β-catenin/TCF4 transcription complex. Indeed, as shown in Fig. 3B, β-catenin binded more P300 when KLF8 was overexpressed in HepG2 cells.

### β-catenin Knockdown Inhibits Proliferation of HepG2 and Hep3B Cells Induced by KLF8 Overexpression

**Figure 4:**
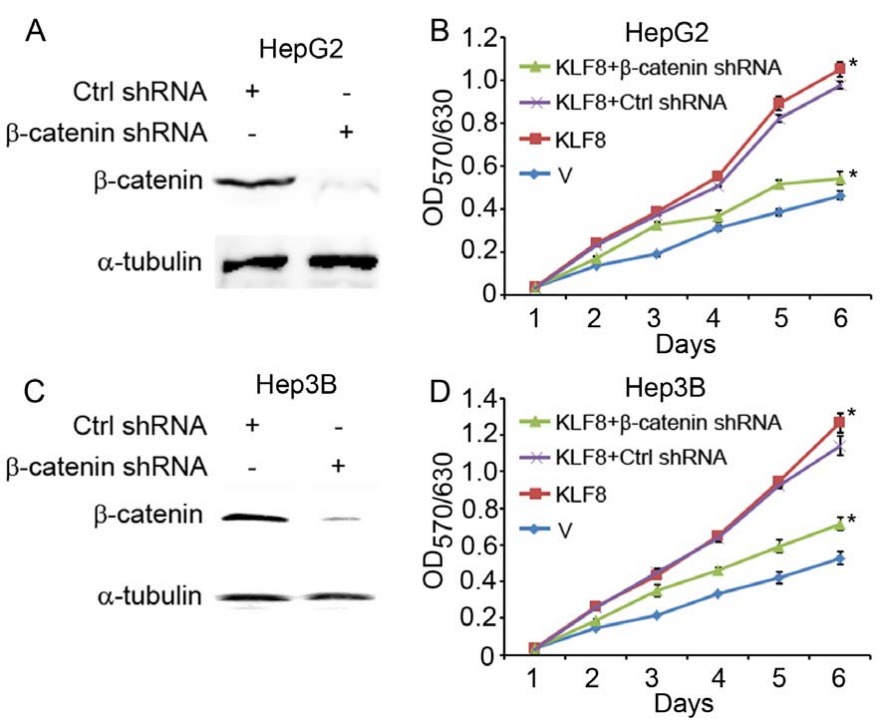
Knockdown of β-catenin inhibits KLF8 induced HCC cell proliferation. (A) β-catenin was knockdown in HepG2/KLF8 cells. HepG2/KLF8 cells were transfected with control shRNA (Ctrl shRNA) or β-catenin shRNA. β-catenin levels were detected by immunostaining with β-catenin antibody, α-tubulin as loading control. (B) In vitro growth of HepG2/V cells, HepG2/KLF8 cells, HepG2/KLF8 cells treated with nonsense control shRNA (Ctrl shRNA) or β-catenin shRNA were measured by MTT assays. (C) β-catenin was knockdown in Hep3B/KLF8 cells. Hep3B/KLF8 cells were transfected with control shRNA (Ctrl shRNA) or β-catenin shRNA. β-catenin levels were detected by immunostaining with β-catenin antibody, α-tubulin as loading control. (D) In vitro growth of Hep3B/V cells, Hep3B/KLF8 cells, Hep3B/KLF8 cells treated with nonsense control shRNA (Ctrl shRNA) or β-catenin shRNA were measured by MTT assays. *, *p*<0.05.

Others’ [13] and our results showed that ectopic expression of KLF8 increased cell proliferation in HepG2 cells (Fig. 4B). To test whether the mechanism that KLF8 regulating Wnt/β-catenin signaling pathway played roles in cell proliferation, we down-regulated the expression of β-catenin in KLF8-overexpressed HepG2 cells using shRNA. Compared to control shRNA, β-catenin-shRNA decreased expression of β-catenin (Fig. 4A), and inhibited the proliferation of KLF8-overexpressed HepG2 cells using MTT assay (Fig. 4B). To further conform the functional role of KLF8/β-catenin in HCC cells, we did the same assay using another HCC cell line Hep3B. As shown in Fig. 4C and 4D, ectopic expression of KLF8 increased Hep3B cells proliferation, β-catenin-shRNA decreased expression of β-catenin and inhibited the proliferation of KLF8-overexpressed Hep3B cells using MTT assay. These results demonstrated that KLF8 regulate HCC cells proliferation through regulating Wnt/β-catenin signaling pathway.

## Discussion

In this report, we showed that KLF8 is a novel targeted gene and regulator in canonical Wnt/β-catenin signaling pathway and this finding is possibly clinically relevant. We identified that KLF8 stabilizes β-catenin, binds with and recruits p300 to β-catenin/TCF4 transcription complex, and activates Wnt/β-catenin target genes transcription. In the meanwhile, activation of Wnt/β-catenin signaling pathway up-regulates both mRNA and protein level of KLF8. Knockdown of β-catenin rescued the phenotype of overexpressed KLF8 HepG2 and Hep3B cells showed that the crosstalk between KLF8 and Wnt/β-catenin signaling pathway plays essential roles in HCC cells proliferation. More importantly, we showed that the aberrant overexpression of KLF8 is strongly correlated to the aberrant nucleus accumulation of β-catenin in human HCC samples, which positively correlates with progressed HCC.

The three major Wnt signaling pathways are involved in almost every aspect of embryonic development and in adult tissue homeostasis. The canonical Wnt/β-catenin signaling pathway governs cell proliferation, differentiation, migration, and invasion. It centers around β-catenin, which binds to TCF/LEF transcription factors and leads to the target genes transcription. In the inactive state, the cytoplasmic β-catenin is continuously degraded. When stimulated by Wnt ligand, β-catenin escapes the degradation, accumulates in the nucleus, binds with TCF transcription complex and induces Wnt target genes expression [1]–[3]. Our study showed that KLF8 is induced by Wnt3a conditioned medium, in the meanwhile KLF8 stabilizes β-catenin in cell cytoplasm and nucleus, enhances Wnt/β-catenin signaling transcription activation. However, the total protein and mRNA level of β-catenin did not change under KLF8 overexpression. These results indicated that KLF8 maybe work on destroying GSK3β/APC/CKIα degradation complex or/and facilitating β-catenin transfer into nucleus. The further studies are needed to demonstrate these hypothesises. Our results also showed that KLF8 binds with TCF4/β-catenin transcription complex, however, whether KLF8 directly binds with β-catenin or/and TCF4, how KLF8 regulate the TCF/β-catenin transcription complex, are still needed to be studied further.

As a transcription factor, KLF8 can negatively regulate the globin and E-cadherin genes through contacting with the C-terminal Binding Protein (CtBP) corepressor, and positively regulate cyclinD1 genes by recruitment of the p300 or p300/CBP associated factor(PCAF) co-activator of the histone acetylase family [9], [14], [15]. Histone acetylase p300 is also needed in TCF 4/β-catenin complex for their transcription activation [16]. Our results showed that KLF8 activates Wnt/β-catenin signaling pathway due to the recruitment of p300 to the β-catenin/TCF complex. Both Wnt/β-catenin signaling pathway and KLF8 can regulate cylinD1 expression [1], [9]. In our system, KLF8 was needed in regulating cyclin D1 gene when Wnt3a conditioned medium added. Since KLF8 can directly bind with the promoter of cyclin D1 [9], there is a possibility that KLF8 may help the β-catenin/TCF transcription complex binding to cyclin D1 promoter. Further studies are needed to explore this question.

HCC is the top 5 common malignant tumors and third cause of cancer-related death [17]. The molecular mechanisms that contribute to tumor initiation or progression of HCC are poorly understood. Our study showed a novel crosstalk between KLF8 and Wnt/β-catenin signaling pathway, which plays important roles in HCC cells proliferation, furthermore, co-overexpression of KLF8 and β-catenin in human HCC samples indicated that KLF8-Wnt/β-catenin signaling pathway may provide novel reasoning for therapy of HCC.
